# Transabdominal Robotic-Assisted Partial Nephrectomy and CT-Guided Percutaneous Cryoablation for the Treatment of De Novo Kidney Tumors After Liver Transplantation

**DOI:** 10.3390/life15020254

**Published:** 2025-02-07

**Authors:** Emanuele Balzano, Lorenzo Bernardi, Gianvito Candita, Arianna Trizzino, Lorenzo Petagna, Elena Bozzi, Paola Scalise, Alessandra Cristaudi, Giovanni Tincani, Daniele Pezzati, Davide Ghinolfi, Laura Crocetti

**Affiliations:** 1 Hepatobiliary Surgery and Liver Transplant Division, Azienda Ospedaliero Universitaria Pisana (AOUP), University of Pisa, 56126 Pisa, Italy; arianna.trizzino@ao-pisa.toscana.it (A.T.); lorenzopetagna@gmail.com (L.P.); d.ghinolfi@ao-pisa.toscana.it (D.G.); 2 Department of Surgery, Lugano Regional Hospital, Ente Ospedaliero Cantonale (EOC), 6900 Lugano, Switzerland; lore.bernardi91@gmail.com (L.B.);; 3 Faculty of Biology and Medicine, University of Lausanne (UNIL), 1015 Lausanne, Switzerland; 4 Interventional Radiology Division, Azienda Ospedaliero Universitaria Pisana, 56126 Pisa, Italyelenabozzi@libero.it (E.B.); paola.scalise@ao-pisa.toscana.it (P.S.); laura.crocetti@unipi.it (L.C.); 5 Department of Surgical, Medical and Molecular Pathology and Critical Care Medicine, University of Pisa, 56126 Pisa, Italy

**Keywords:** robotic partial nephrectomy, cryoablation, de novo kidney tumors, liver transplantation, immunosuppression

## Abstract

The management of de novo kidney tumors (DKTs) after liver transplantation (LT) is challenging due to previous transplant surgery and calcineurin inhibitors (CNI)-related nephrotoxicity. Minimally invasive renal-sparing strategies like robot-assisted partial nephrectomy (RPN) are favored, but a transperitoneal approach may be limited by the previous transplant surgery and the location of the DKT; in such cases, CT-guided cryoablation may be an alternative option. In this retrospective cohort study, we aimed to compare RPN and cryoablation for the treatment of DKT in LT recipients. The primary endpoints were the efficacy (R0 resection in RPN, absence of the tumor at first follow-up for cryoablation) and the safety of the procedures (postoperative morbidity and increase in creatine level). The periprocedural costs and the oncologic efficacy (recurrence and overall survival) were the secondary endpoints. Twelve LT recipients (91.7% males, mean age 65 years) underwent RPN (n = 6) or cryoablation (n = 6) for DKT; the median interval between LT and diagnosis of DKT was 142.5 vs. 117.5 months, respectively. Efficacy was obtained in all patients after RPN and cryoablation. Postoperative morbidity was 16.7% in each group, and the postoperative increase in creatinine values was similar. Hospital stay was shorter following cryoablation vs. RPN (3.1 vs. 6.7 days; *p* = 0.03). The mean procedural costs were higher for RPN. There was no mortality and none of the patients had signs of recurrence after a median follow-up of 40.5 months. Both RPN and CT-guided cryoablation were safe and effective for the treatment of selected patients with DKT after LT. When applicable, cryoablation may be cost-effective and provide faster recovery.

## 1. Introduction

De novo cancers develop with a 10-fold increased incidence in liver transplant (LT) recipients compared to the general population and account for almost 30% of deaths 10 years after transplantation [1,2,3,4]. Several risk factors for de novo tumors following LT have been identified, including diabetes, immunosuppressive-related metabolic disorders, smoking history, etiology of the liver disease, indicating LT (i.e., alcohol-related cirrhosis), and type of immunosuppression [5,6,7]. While tailored strategies to mitigate the incidence of de novo cancers after LT are urgently needed, their treatment remains a major concern for the transplant community [8,9]. Although de novo kidney tumors (DKTs) represent a rare entity among de novo cancers in LT recipients, their management may be particularly challenging due to the presence of chronic kidney disease (CKD) related to the use of calcineurin inhibitor-based immunosuppression (CNI) and to the presence of post-transplant adhesions [5,10,11]. 

Minimally invasive partial nephrectomy (PN), aiming to preserve kidney function as much as possible, is the treatment of choice for early-stage kidney tumors [12,13]. However, post-transplant adhesions could limit the applicability of minimally invasive transabdominal approaches. Among minimally invasive techniques for PN, robotic partial nephrectomy (RPN) is ensured to overcome some of the limitations of laparoscopy, easing maneuvers requiring special dexterity such as renorrhaphy or the isolation of the renal pedicle for hilar clamping [14,15,16]. Furthermore, the robotic approach is emerging as a tool used to increase the feasibility of redo-surgery in patients with previous major abdominal interventions like liver resections or LT potentially needing extensive adhesiolysis [17,18,19]. In parallel, CT-guided percutaneous cryoablation is an alternative, non-surgical option for the treatment of small kidney tumors in selected patients like frail ones, those with already compromised renal function, or those declining surgery [13,20]. Cryoablation recently showed the potential to fasten the recovery and lower periprocedural costs while guaranteeing similar outcomes compared to RPN [21,22].

The current study aimed to address the efficacy and safety of RPN and cryoablation for the treatment of DKT in LT recipients, in which it—we hypothesize—could play a complementary role.

## 2. Materials and Methods

### 2.1. Study Design and Setting

This was a retrospective study including patients diagnosed with DKT after LT that underwent RPN or CT-guided cryoablation at the University Hospital of Pisa in the period from January 2016 (beginning of RPN and cryoablation activity) to December 2023. All the patients included had prior LT in the same institution. The liver transplant program at the University Hospital of Pisa started in January 1996, and 2786 patients underwent LT up to December 2023. The primary endpoint of this study was the technical efficacy (defined as R0 margin for RPN and absence of viable tumor at first follow-up imaging for cryoablation) and the safety of the procedures (intra- and postoperative complications, modifications of the creatine levels compared to the baseline). Procedure-related costs and oncologic efficacy (recurrence and survival) were the secondary endpoints. This study followed the STROBE guidelines for reporting observational cohort studies [23].

### 2.2. Participants

Inclusion criteria: all LT recipients who underwent RPN or cryoablation for the treatment of DKT at the University Hospital of Pisa in the period of January 2016–December 2023.

Exclusion criteria: extra-renal tumor spread.

### 2.3. Data Source and Outcome

Data extracted from the institutional LT database and from electronic or paper charts of the patients included age, sex, BMI, year and indication of prior LT, etiology of liver disease before LT, immunosuppressive regimen, presence of CKD or creatinine values before receiving RPN or cryoablation, delay between diagnosis of DKT and LT, size and anatomical location of DKT, stage, and RENAL nephrometry score [12,24]. The outcomes of RPN and cryoablation included operative time, technical efficacy, intra- and postoperative morbidity according to the Clavien–Dindo classification, creatinine levels following the procedures, and length of hospital stay [25]. Procedure-related costs (EUR) included the costs of both the procedure and of the hospital stay.

### 2.4. Indications to Surgery or Cryoablation

The indication to proceed with either RPN or cryoablation was validated by a multidisciplinary team (MDT) discussion including surgeons, radiologists and interventional radiologists, oncologists, nephrologists, and hepatologists. The diagnosis of DKT was based on imaging (CT scan and/or abdominal MRI). Pathology confirmation (biopsy) was not mandatory before proceeding to treatment as a CT examination negative for kidney tumors (acquired during pre-LT evaluation) was available for all patients. The feasibility of a minimally invasive surgical approach (RPN) vs. CT-guided cryoablation was discussed based on a CT scan study of the anatomy and characteristics of the patients. RPN was favored in the case of anterior location, the interposition of neighbor organs (i.e., the colon, duodenum) limiting the reachability of the kidneys, or proximity to inferior vena cava. A percutaneous radiological approach (cryoablation) was considered in older or more fragile patients, a lateral or posterior location, or more advanced CKD.

### 2.5. Procedures and Follow-Up

The technical details of RPN (port placement, patient positioning, instrument used) as well as of CT-guided cryoablation are detailed in the Supplementary Materials. RPN was performed by senior hepatobiliary and transplant surgeons with experience in robotic general and hepatobiliary surgery [26,27]. Cryoablations were performed by senior interventional radiologists dedicated to interventional oncology. Follow-up following LT was according to the EASL guidelines [28]. After RPN, patients were followed by contrast-enhanced imaging (CT or MRI) at 1 month, 3 months, and 6 months, then every 6 months for the first 2 years, and then annually. In patients with eGFR (estimated glomerular filtration rate) lower than 35 mL/min, contrast-enhanced ultrasound (CEUS) was performed. Together with imaging, blood and urine tests were obtained. After cryoablation, the follow-up protocol was identical, with the first imaging (1 month) used to confirm the efficacy of the CT-guided cryoablation (absence of residual tumor). Persistence and local recurrence were defined as the presence of nodular enhancing tissue at the first follow-up imaging or at necrotic margins after a complete ablation, respectively. 

### 2.6. Statistical Analysis

According to their level of measurement and distribution, continuous variables were expressed as means and standard deviations (SDs) or medians and interquartile ranges (IQRs), while categorical variables were described as frequencies. Data were compared with the t-test for continuous values with normal distribution, the Mann–Whitney U test for continuous values without normal distribution, and Pearson’s chi-square or Fisher’s exact tests for categorical values. The level of significance was set at 5%. Survival was censored at death or the latest follow-up. Statistical analysis was performed by IBM SPSS Statistics™ (v29.0).

## 3. Results

### 3.1. Participants

**Figure 1:**
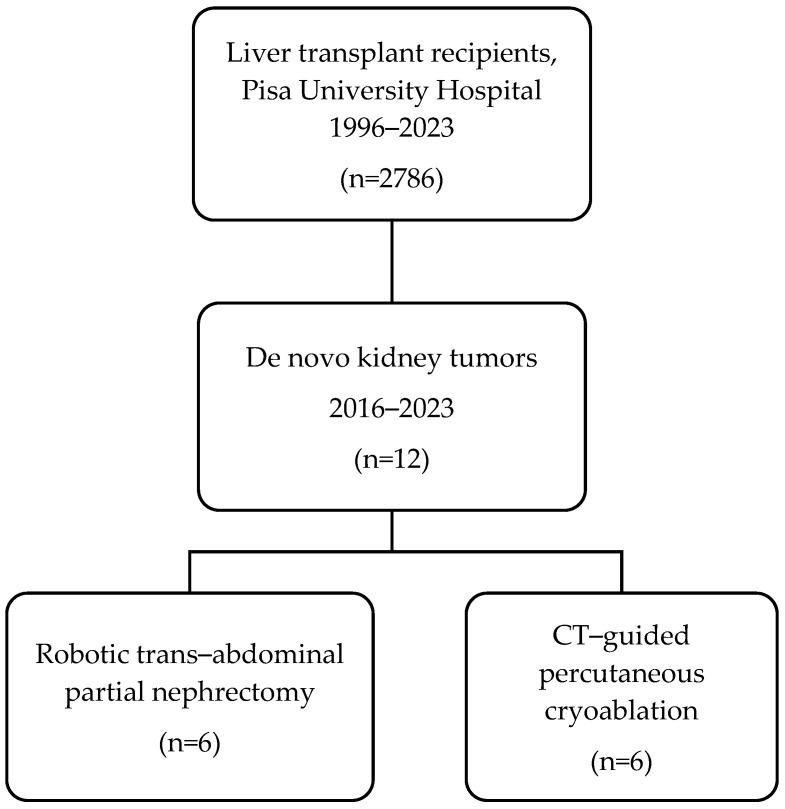
Flowchart of this study.

Twelve LT recipients (11 males, 92%) underwent RPN (n = 6) and cryoablation (n = 6) for DKT in the study period (Figure 1). The mean age was 61.0 vs. 68.7 years (*p* = 0.07) and the mean BMI was 25.6 vs. 23.4 kg/m^2^ (*p* = 0.69). The median interval between LT and diagnosis of DKT was 142.5 vs. 117.5 months (*p* = 0.57) in RPN vs. cryoablation, respectively. DKT stage was cT1a in five (83.3%) vs. cT1b in one (16.7%) in both RPN and cryoablation groups. DKTs were in the anterior aspect of the kidney in three (50%) patients who underwent RPN vs. none in the cryoablation group. All patients that underwent cryoablation had posteriorly located DKT. None of the patients had a preoperative tumor biopsy. The characteristics of the participants are detailed in Table 1.

### 3.2. Efficacy and Safety

**Figure 2:**
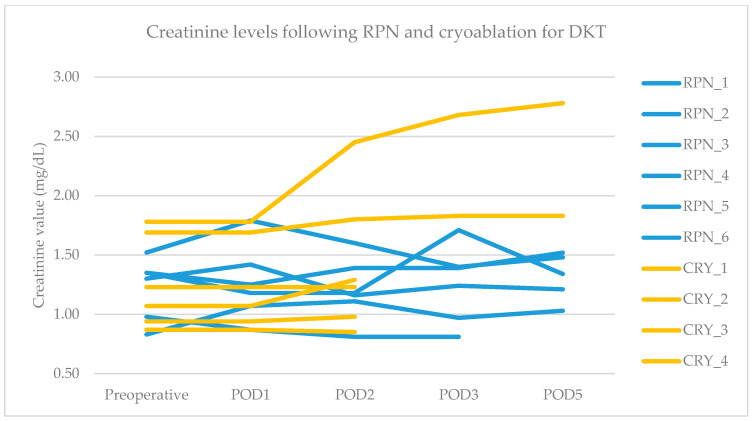
Pre- and postoperative creatinine values in RPN and cryoablation groups. Legend: RPN, robotic partial nephrectomy; CRY, cryoablation; POD, postoperative day.

**Figure 3:**
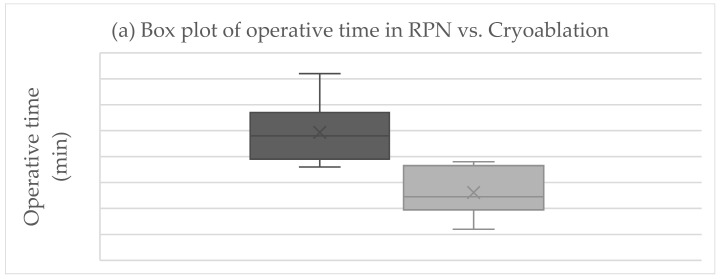

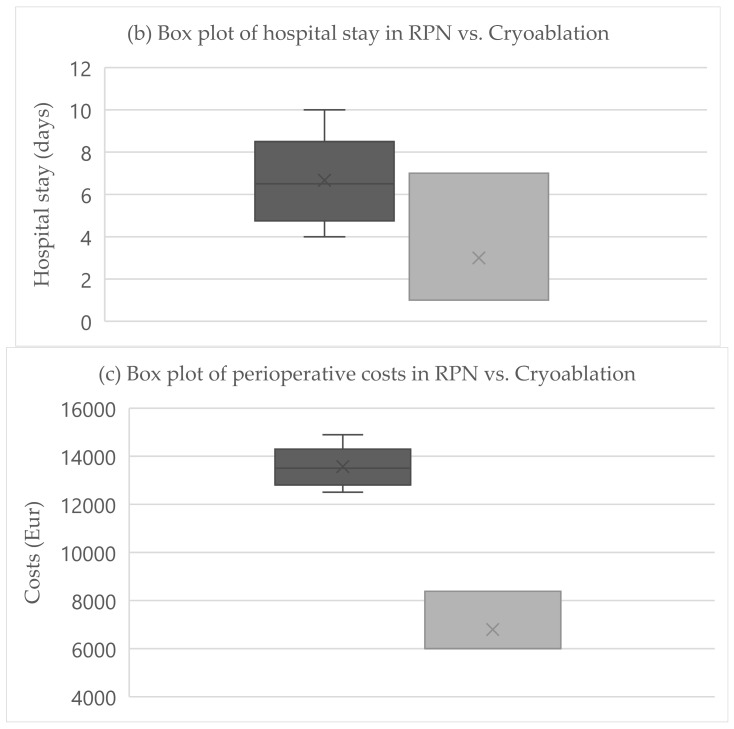
Boxplots of (**a**) operative time, (**b**) hospital stay, (**c**) costs in RPN, and cryoablation. Legend: RPN, robotic partial nephrectomy. Middle lines depict median values, the ends of the boxes depict first and third quartiles, whiskers indicate minimum and maximum values, and “x” indicates the mean value.

The primary endpoint was obtained in all patients after RPN and cryoablation. No cases of viable tumor persistence were detected. In the RPN group, one patient experienced intraoperative complications (uncontrolled bleeding requiring conversion to open surgery) vs. none in the cryoablation group. The postoperative morbidity rate was 16.7% after both RPN and cryoablation (one patient in each group, both grade II complications according to Clavien–Dindo), while major morbidity was not observed (Table 2). The creatinine levels remained stable, except in a patient in the cryoablation group who developed acute kidney injury of grade II (Figure 2). Operative time was longer for RPN compared to cryoablation (Figure 3a). Hospital stay was longer following RPN (6.7 vs. 3.1 days in cryoablation group; *p* = 0.03), as shown in Figure 3b. The patient who experienced intraoperative bleeding requiring conversion to laparotomy had a hospital stay of 10 days. Procedure-related mortality was nihil in the series.

### 3.3. Costs

Mean procedure-related costs (EUR) per patient were nearly doubled in the RPN compared to cryoablation groups (13,586.0 vs. 6792.0 Eur; *p* = 0.01) (Table 2, Figure 3c).

### 3.4. Oncologic Efficacy (Recurrence and Survival)

**Figure 4:**
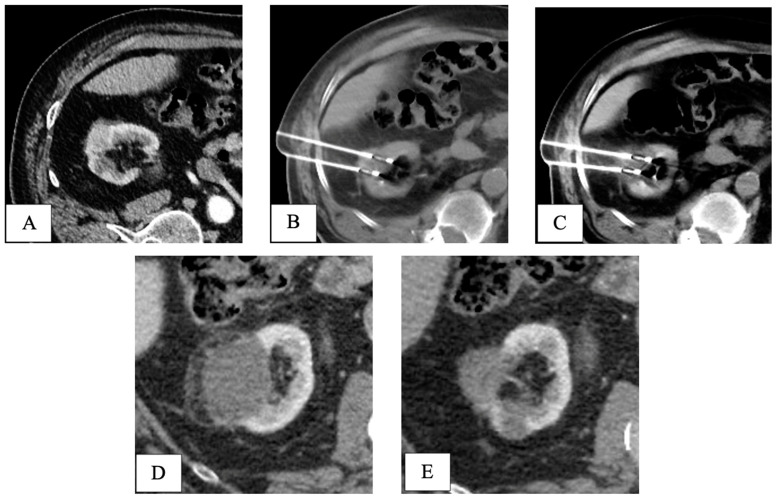
CT-guided cryoablation of de novo kidney tumor in a patient who underwent liver transplant 5 years before for HCV-related HCC. Legend: (**A**) preliminary contrast-enhanced CT scan confirmed the presence of a T1a DKT sizing 2cm in the right kidney; (**B**) under CT guidance, 2 cryoprobes were placed within the renal lesion; (**C**) control CT scan showed the presence of a hypodense ice-ball, including all the volume of the nodule to be treated. (**D**) One-month follow-up CT scan detected complete response, with a hypodense cryo-induced ablation area; (**E**) 3-year follow-up CT scan demonstrated prolonged response, with ablation area decreasing in size. DKT, de novo kidney tumor.

Final pathology following RPN revealed five pT1 papillary-type renal cell carcinomas and one fibrotic nodule without any sign of cancer. At a mean follow-up of 40.5 months, all patients in both groups were alive without signs of recurrence (Table 2). An example of the oncologic efficacy of CT-guided cryoablation is showed in Figure 4.

## 4. Discussion

Both RPN and cryoablation were safe and effective for the treatment of selected patients with DKT after LT. At the price of reasonable morbidity rates and no postoperative mortality, efficacy was obtained in all the patients (surrogated by R0 resection in RPN and absence of viable tumor at the first follow-up imaging following cryoablation, respectively). Importantly, none of the patients in the RPN group required conversion to laparotomy due to previous transplant-related adhesions. In one case at the beginning of our experience (case number 2, left-sided DKT), urgent laparotomy was necessary due to difficult hemostasis for bleeding from the kidney parenchyma. Hemostasis was obtained without the need to convert the procedure to radical nephrectomy. These results support the safe use of the robotic approach in patients previously treated by open transplant surgery, allowing us to extend the benefit of a minimally invasive approach to this subset of patients. This is a point that has been increasingly studied in recent years, like for minimally invasive liver resections following previous open hepatectomy or liver transplantation [17,18,19].

Although reserved for slightly different patients (older, posterior DKT), CT-guided cryoablation showed similar results compared with RPN but a more favorable profile as per hospital stay and periprocedural costs, suggesting its valuable role as an alternative to the robotic approach for PN in selected patients.

In the present series, none of the LT recipients had preoperative biopsy before receiving RPN or cryoablation, and in one patient in the RPN group (16.7%), pathology did not show a malignant tumor. Although a consensus on this subject is lacking, renal mass biopsy before surgery for clinical T1a tumors is usually recommended, as up to 30% are benign and may not need an intervention [13]. The omission of pre-emptive histologic confirmation followed MDT discussion and was based on highly suspicious imaging in the context of long-term immunosuppression in patients who had a CT examination negative for kidney tumors (acquired during pre-LT evaluation). If pre-emptive biopsy is avoided, RPN would still allow for a final histologic diagnosis, while cryoablation would not. If cryoablation is performed, pre-emptive histologic confirmation of renal cell carcinoma would be particularly important to tailor postprocedural follow-up and ensure adequate information to patients.

To the best of our knowledge, there are no studies focused on RPN or cryoablation for DKT in LT recipients. The management of DKT in LT recipients basically relies on the same principles of the management of renal cell carcinoma in non-transplanted patients; thus, some indirect comparisons could be made with similar studies including non-LT patients. Our findings aligned with a few previous investigations reporting a longer operative time and hospital stay for RPN compared with cryoablation with similar short- and long-term outcomes. RPN was generally adopted for younger patients with larger tumors and a higher RENAL score [29]. Costs were always in favor of cryoablation [21,22]. Interestingly, the anatomical location (anterior/posterior) of the kidney tumor was not systematically reported, and its relationship with the type of treatment (RPN or cryoablation) could not be addressed.

Some limitations of this study warrant discussion. First, this was a retrospective analysis including a few selected patients treated in a high-volume transplant center and with limited follow-up. Accordingly, this study was prone to selection bias and our findings/conclusions might not be directly translated to smaller facilities or generalizable. Moreover, DKTs represent rare entities among de novo tumors in LT recipients [1,2]. Because of the small sample size, we were unable to catch neither the potential impact of the different immunosuppressive regiments on the development of DKT nor on the results of each technique. Second, this series included only T1 kidney tumors since these patients were all under close follow-up after LT. The efficacy and safety of both RPN and cryoablation for larger tumors in LT recipients remain to be explored. Finally, a formal comparison with other minimally invasive techniques like transabdominal laparoscopic PN, laparoscopic-assisted cryoablation, or retroperitoneal RPN would be interesting [30,31]. Due to the simultaneous implementation of both laparoscopic and robotic programs in our division in 2014, the rarity of DKT in LT recipients, and the expected advantages of robotics compared to laparoscopy for PN, these cases were selected for the robotic transabdominal approach directly [32]. Besides image-guided cryoablation, even radiofrequency and microwave ablations have been demonstrated to be safe and effective in treating kidney tumors [33,34,35]. According to the European Urology Association (EUA) guidelines, local therapies like cryoablation can be used for tumors up to 4 cm [12]. At our institution, the preferred locoregional approach for treating kidney tumors is CT-guided cryoablation due to the unique possibility of monitoring the ablation zone though the visualization of the hypodense “ice-ball” during treatment.

This study provided valuable insights and supported clinical decision-making on a rare condition for LT recipients. The correct selection of patients to one approach or the other is pivotal, since both techniques have the potential to provide good short-term and excellent long-term results; RPN and cryoablation should be considered complementary techniques rather than mutually exclusive. Both should follow pre-emptive histologic confirmation through DKT biopsy with the objective of avoiding unnecessary interventions and (for cryoablation) tailoring postprocedural surveillance. Given its cost-effectiveness and the potential for rapid recovery, cryoablation could be used in anatomically favorable situations and even in fragile patients proceeded by biopsy. On the other hand, RNP could be the choice in the case of anatomically unfavorable locations for percutaneous approaches. 

## 5. Conclusions

Both RPN and cryoablation were safe and effective for the treatment of selected patients with early-stage DKT after LT. When applicable, cryoablation may be cost-effective and provide faster recovery. Based on our experience in T1 DKT, CT-guided cryoablation preceded by biopsy may be the first choice. If cryoablation is not deemed feasible or safe (i.e., due to DKT location), RPN could be used. Larger studies in a prospective setting are needed to confirm these preliminary results.

## Figures and Tables

**Table 1:** Baseline characteristics of patients included.

	RPN (n = 6)	Cryoablation (n = 6)	*p*
**Sex male, n (%)**	5 (83.3)	6 (100)	1.0
**Age (years), mean (SD)**	61.0 (4.3)	68.7 (8.4)	0.07
**BMI (kg/m^2^), mean (SD)**	25.6 (1.6)	23.4 (1.3)	0.69
**Liver disease prior to LT, n (%)**			
HBV/HCV	5 (83.3)	5 (83.3)	1.0
OH	1 (16.7)	0 (0)	
PSC	0 (0)	1 (16.7)	
**Indication to LT, n (%)**			
Advanced cirrhosis	4 (66.7)	3 (50.0)	0.64
HCC	1 (16.7)	3 (50)	
Acute hepatitis	1 (16.7)	0 (0)	
**Interval between LT and DKT diagnosis (months), median (IQR)**	142.5 (107.5–180.5)	117.5 (62.5–180.0)	0.57
**Immunosuppressive regime at DKT diagnosis, n (%)**			
Tacrolimus	3 (50)	3 (50)	1.0
Everolimus	1 (16.7)	2 (33.3)	
Ciclosporin	2 (33.3)	0 (0)	
Mycophenolate	0 (0)	1 (16.7)	
**DKT size (mm), mean (SD)**	25.3 (10.6)	23.8 (10.3)	0.81
**Side of DKT, n (%)**			
Right	3 (50)	1 (16.7)	0.47
Left	3 (50)	5 (83.3)	
**Location of DKT, n (%)**			
Polar superior	1 (16.7)	1 (16.7)	0.92
Polar inferior	4 (66.7)	3 (50)	
Meso-renal	1 (16.7)	2 (33.3)	
Anterior	3 (50)	0 (0)	NA
Posterior	3 (50)	6 (100)	
**Pre-procedural biopsy, n (%)**	0 (0)	0 (0)	NA
**RENAL score [24], mean (SD)**	7.4 (2.2)	5.5 (1.2)	0.10
**T stage, n (%)**			
T1a	5 (83.3)	5 (83.3)	1.0
T1b	1 (16.7)	1 (16.7)	
**Baseline creatinine value (mg/dL), mean (SD)**	1.26 (0.31)	1.26 (0.38)	1.0

**Abbreviations:** RPN, robotic partial nephrectomy; LT, liver transplant; HBV, hepatitis B virus; HCV, hepatitis C virus; OH, alcohol; PSC, primary sclerosing cholangitis; HCC, hepatocellular carcinoma; DKT, de novo kidney tumors; SD, standard deviation; IQRs, interquartile ranges.

**Table 2:** Intra- and postoperative results, costs, and long-term results of RPN and cryoablation.

	RPN (n = 6)	Cryoablation (n = 6)	*p*
**Operative time (min), mean (SD)**	247 (63.0)	131 (48)	**0.01**
**Intraoperative complications, n (%)**	1 (16.7)	0 (0)	NA
Conversions to open surgery, n (%)	1 (16.7)	–	
**Postoperative morbidity (CD), n (%)**	1 (16.7)	1 (16.7)	1.0
**Postoperative major morbidity (CD III or more), n (%)**	0 (0)	0 (0)	NA
**Hospital stay (days), mean (SD)**	6.7 (2.2)	3.1 (3.0)	**0.03**
**Pathology, n (%)**			
papillary type renal cell carcinoma	5 (83.3)	–	
negative for malignancy	1 (16.7)	–	
**Technical efficacy, n (%)**	6 (100)	6 (100)	1.0
R0 margin	6 (100)	–	
Absence of viable tumor at 1st follow-up imaging	–	6 (100)	
**Procedure-related mortality, n (%)**	0 (0)	0 (0)	
**Periprocedural costs (EUR) per patient, mean (SD)**	13,586.0 (860.0)	6792.0 (1230.0)	**0.01**
**Follow-up duration (months), median (IQR)**	49.5 (41.5–55.7)	20.5 (12.2–38.5)	**<0.001**
**Patients alive at last follow-up, n (%)**	6 (100)	6 (100)	1.0
**Recurrence, n (%)**	0 (0)	0 (0)	NA

**Abbreviations:** RPN, robotic partial nephrectomy; SD, standard deviation; IQRs, interquartile ranges.

## Data Availability

The anonymized dataset used in this study is available upon reasonable request to the corresponding author.

## Supplementary Materials

The following supporting information can be downloaded at: https://www.mdpi.com/article/10.3390/life15020254/s1, Technical details of RPN and cryoablation (word file).
