# Supplementary material for: Transabdominal Robotic-Assisted Partial Nephrectomy and CT-Guided Percutaneous Cryoablation for the Treatment of De Novo Kidney Tumors After Liver Transplantation
Source: Life (Basel). 2025 Feb 7;15(2):254. doi: 10.3390/life15020254 (PMC11856640; doi:10.3390/life15020254)
Supplement: Supplementary file 1 [file life-15-00254-s001.zip › life-3443561-supplementary.docx]

**Supplementary material (1)**

**Technical details of RPN and cryoablation (word file).**

*Robotic partial nephrectomy (RPN)*

The patient is placed in semi lateral decubitus (contralateral to the DKT). Once pneumoperitoneum through an “open-laparoscopy” technique is achieved, the camera is inserted; the abdominal cavity is inspected looking for injuries or adhesions. Insertion of the remaining robotic instruments (fenestrated bipolar, monopolar scissors, Pro- Grasp) follows under direct view. A total of three-to-four 8 mm robotic ports are used. Perirenal adhesions are carefully divided by using monopolar scissors. The white line of Toldt is divided and the colon is retracted medially to expose the Gerota’s fascia, the ureter is identified and isolated with a vessel loop. Next, the renal pedicle is identified, fully exposed, and mobilized. The perirenal fat is gently elevated from the kidney capsule to create wide exposure. Once the nodule is identified, the ultrasound probe is introduced to clarify the depth and margins of the nodule. The line of parenchymal dissection is scored with the cautery. To avoid the risk of bleeding, bulldog clamps are introduced and passed to the Pro-Grasp. All major arterial branches are clamped and then the tumor is completely excised and placed above the liver or spleen. Clips, cautery or renorrhaphy are used to control bleedings. Once hemostasis is assured, the bulldog clamps are released. The tumor is placed in an endo-bag and extracted from umbilical port access. A drainage tube is placed in the lateral renal fossa. Closing of the abdominal accesses follows.

*Cryoablation*

The argon-based CryoCare Touch™ cryoablation system (Varian, Siemens, Palo Alto, CA) was used, with one or more cryoprobes of different sizes according to the tumor size. A preliminary CT of the abdomen allows to identify the shape, size, location, and anatomical relationship of DKT, then the patient is positioned accordingly (prone, supine, lateral decubitus). The number and caliber (1.7 mm or 2.4 mm) of the probes are chosen according to lesion size and actual probe position, to cover all the nodule plus a margin of 5mm. Probes are positioned under CT-guidance. If bowel loops are in the immediate proximity of the lesion to be treated adjunctive 21G Chiba needles are used to inject saline mixed with iodinated contrast media (2%) to displace and protect the organ from ice damage (“hydro dissection” maneuver). Two cycles of freezing are interspersed with a passive thawing cycle; finally, after active thawing with helium, probes are removed. During procedure monitoring is performed by acquiring non enhanced CT that shows the hypodense ice-ball. The margin of the ice-ball corresponds to the isotherm of 0°C. Because cytotoxic temperatures in the kidney are below -20°C it is estimated that the hypodense ice-ball should exceed the intended area of ablation of at least 4 mm.
